# Tumor necrosis factor inhibition attenuates white matter gliosis after systemic inflammation in preterm fetal sheep

**DOI:** 10.1186/s12974-020-01769-6

**Published:** 2020-03-23

**Authors:** Robert Galinsky, Simerdeep K. Dhillon, Justin M. Dean, Joanne O. Davidson, Christopher A. Lear, Guido Wassink, Fraser Nott, Sharmony B. Kelly, Mhoyra Fraser, Caroline Yuill, Laura Bennet, Alistair Jan Gunn

**Affiliations:** 1 Department of Physiology, Faculty of Medical and Health Sciences, University of Auckland, Private bag 92019, Auckland, 1023 New Zealand; 2 The Ritchie Centre, Hudson Institute of Medical Research, Clayton, Victoria Australia; 3 Department of Obstetrics and Gynaecology, Monash University, Melbourne, Victoria Australia

**Keywords:** TNF, Etanercept, Neuroprotection, Preterm infant, Inflammation, Brain, Anti-inflammatory

## Abstract

**Background:**

Increased circulating levels of tumor necrosis factor (TNF) are associated with greater risk of impaired neurodevelopment after preterm birth. In this study, we tested the hypothesis that systemic TNF inhibition, using the soluble TNF receptor Etanercept, would attenuate neuroinflammation in preterm fetal sheep exposed to lipopolysaccharide (LPS).

**Methods:**

Chronically instrumented preterm fetal sheep at 0.7 of gestation were randomly assigned to receive saline (control; *n* = 7), LPS infusion (100 ng/kg i.v. over 24 h then 250 ng/kg/24 h for 96 h plus 1 μg LPS boluses at 48, 72, and 96 h, to induce inflammation; *n* = 8) or LPS plus two i.v. infusions of Etanercept (2 doses, 5 mg/kg infused over 30 min, 48 h apart) started immediately before LPS-exposure (*n* = 8). Sheep were killed 10 days after starting infusions, for histology.

**Results:**

LPS boluses were associated with increased circulating TNF, interleukin (IL)-6 and IL-10, electroencephalogram (EEG) suppression, hypotension, tachycardia, and increased carotid artery perfusion (*P* < 0.05 vs. control). In the periventricular and intragyral white matter, LPS exposure increased gliosis, TNF-positive cells, total oligodendrocytes, and cell proliferation (*P* < 0.05 vs control), but did not affect myelin expression or numbers of neurons in the cortex and subcortical regions. Etanercept delayed the rise in circulating IL-6, prolonged the increase in IL-10 (*P* < 0.05 vs. LPS), and attenuated EEG suppression, hypotension, and tachycardia after LPS boluses. Histologically, Etanercept normalized LPS-induced gliosis, and increase in TNF-positive cells, proliferation, and total oligodendrocytes.

**Conclusion:**

TNF inhibition markedly attenuated white matter gliosis but did not affect mature oligodendrocytes after prolonged systemic inflammation in preterm fetal sheep. Further studies of long-term brain maturation are now needed.

## Background

Preterm birth is associated with long-term neurodevelopmental impairments, such as cerebral palsy, for which the combined lifetime economic cost in the USA was estimated to be over USD11.5 billion in 2003 [1]. The cause of neurodevelopmental impairment after preterm birth is multifactorial. However, there is compelling evidence that exposure to perinatal infection/inflammation is strongly associated with preterm birth and impaired neurodevelopment [2–4]. In recent studies, long-term neurodevelopmental impairment was associated with diffuse injury in the white matter tracts, with evidence of chronic gliosis and impaired oligodendrocyte maturation, but limited cell loss [5, 6]. These maturational disturbances are considered to be key factors responsible for the reduced white and gray matter volumes, and neurobehavioral disturbances and intellectual disabilities, seen in recent cohorts of preterm infants [7, 8].

The cause of neuroinflammation and impaired cellular maturation is multifactorial [4, 9]; however, pro-inflammatory cytokines such as tumor necrosis factor (formerly known as tumor necrosis factor-alpha) are upregulated within the white matter at post-mortem in preterm infants with periventricular leucomalacia [10, 11] and in preterm large animal models of perinatal inflammation [12, 13]. Elevated cord blood levels of tumor necrosis factor (TNF) are strongly associated with impaired neuronal activity in the first few days of life [14] and developmental delay at 2 years of age [15]. Supporting these data, large animal studies consistently show that TNF is one of the principal pro-inflammatory cytokines that is acutely upregulated in the circulation after the induction of intrauterine inflammation [16–18]. In neonatal rodents, systemic TNF inhibition after exposure to a combination of systemic inflammation and ibotenate-induced excitotoxicity was associated with a reduction in the severity of gliosis, apoptosis, and brain lesion size [19]. Furthermore, in vitro studies show that TNF inhibits oligodendrocyte development [20], and that oligodendrocyte maturation can be restored with TNF inhibition [21]. Critically, in P3 rats, TNF inhibition immediately after LPS injection reduced microgliosis and astrocytosis, and attenuated the apoptotic loss of oligodendrocyte precursor cells [22]. Collectively, these data raise the possibility that systemic infiltration and/or local production of TNF plays a key role in the pathogenesis of impaired neurodevelopment, and thus, that TNF inhibition may be a viable therapeutic target for reducing the severity of inflammation-induced brain injury in preterm infants.

In this study, we tested the hypothesis that systemic TNF inhibition, using the clinically available, soluble, and highly specific TNF antagonist, Etanercept, would reduce the severity of neuroinflammation and brain injury in 0.7 gestation preterm fetal sheep exposed to lipopolysaccharide-induced inflammation. At this age, brain development in fetal sheep is broadly equivalent to the 28- to 30-week human infant [23].

## Materials and methods

All procedures were approved by the Animal Ethics Committee of The University of Auckland under the New Zealand Animal Welfare Act, and the Code of Ethical Conduct for animals in research established by the Ministry of Primary Industries, Government of New Zealand. Eighteen Romney/Suffolk fetal sheep underwent aseptic surgery between 97 and 99 days gestation (term = 147 days). Food but not water was withdrawn 18 h before surgery. Ewes were given long-acting oxytetracycline (20 mg/kg, Phoenix Pharm., Auckland, New Zealand) i.m. 30 min before the start of surgery. Anesthesia was induced by i.v. injection of propofol (5 mg/kg; AstraZeneca Limited, Auckland, New Zealand) and maintained using 2–3% isoflurane in O_2_ (Bomac Animal Health, NSW, Australia). During surgery, ewes received an i.v. infusion of isotonic saline (250 mL/h) to maintain fluid balance. Depth of anesthesia, maternal heart rate, and respiration were continuously monitored by trained anesthetic staff.

### Instrumentation

In brief, following a maternal midline abdominal incision, the fetus was exposed, and polyvinyl catheters were inserted in the left femoral and brachial arteries, brachial vein, and amniotic cavity. Vascular flow probes (Transonic Systems, Ithaca, NY, USA) were placed around the right femoral artery (2.5 mm) and right carotid artery (3 mm) to monitor femoral and carotid arterial blood flows (FBF and CaBF, respectively). A pair of electrodes was sewn over the fetal chest to measure the fetal electrocardiogram (ECG). Two pairs of electroencephalograph (EEG) electrodes (AS633-7SSF; Cooner Wire, Chatsworth, CA, USA) were placed through burr holes onto the dura over the parasagittal parietal cortex (5 and 10 mm anterior to bregma and 5 mm lateral) and secured with cyanoacrylate glue. To measure cortical impedance, a pair of electrodes (AS633-3SSF; Cooner Wire) was placed over the dura 5 mm lateral to the EEG electrodes. A pair of electrodes was sewn into the nuchal muscle to record electromyographic (EMG) activity to measure fetal movement and a reference electrode was sewn over the occiput. All fetal leads were exteriorized through the maternal flank. Antibiotics (Gentamicin 80 mg; Rousell Ltd., Auckland, New Zealand) were administered into the amniotic sac before the closure of the uterus. A maternal long saphenous vein was catheterized to provide access for postoperative care.

Sheep were housed in separate metabolic cages with access to water and food *ad libitum* in a temperature-controlled room (16 ± 1 °C, humidity 50 ± 10%) with a 12:12 h light/dark cycle. Five days of postoperative recovery was allowed before experiments commenced. During this time, ewes received intravenous antibiotics daily for 4 days (benzylpenicillin sodium 600 mg, Novaris, Auckland, New Zealand and Gentamycin 80 mg). Fetal catheters were maintained patent by continuous infusion of heparinized saline (20 IU/mL) at a rate of 0.2 mL/h.

### Experimental recordings

Fetal mean arterial blood pressure (MAP), corrected for maternal movement by subtraction of amniotic pressure, FBF, CaBF, ECG, EEG, impedance and nuchal EMG were recorded continuously for offline analysis using custom data acquisition software (LabView for Windows, National Instruments, TX, USA). The blood pressure signal was collected at 64 Hz and low-pass filtered at 30 Hz. The fetal ECG was analog filtered between 0.05 and 100 Hz and digitized at 512 Hz, and used to derive fetal heart rate (FHR). The analog EEG signal was low-pass filtered with the cut off frequency at 500 Hz, digitized at a sampling frequency of 1024 Hz, then further low-pass filtered (in software) with the low-pass cut-off at 128 Hz, and downsampled to 256 Hz. The intensity (power) was derived from the intensity spectrum signal between 0.5 and 20 Hz, while the spectral edge was calculated as the frequency below which 90% of the intensity was present [24]. For data presentation, total EEG power was normalized by log transformation (dB, 10 × log intensity). The nuchal EMG signal was band-pass filtered between 100 Hz and 1 kHz, and then integrated using a time constant of 1 s and digitized at 512 Hz.

### Experimental protocol

**Fig. 1:**
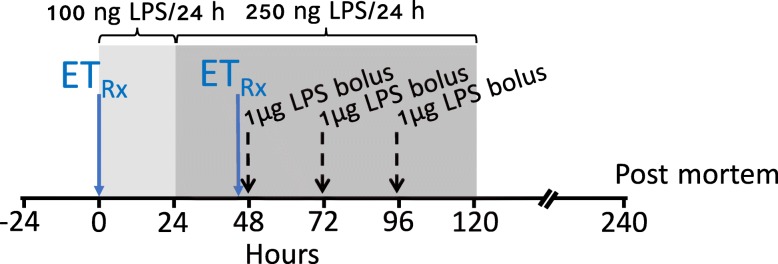
Schematic outlining the study design. The study consisted of 3 groups: control (*n* = 7), LPS (*n* = 8), and Etanercept (ET) + LPS (*n* = 8). The light shaded area represents the period of low dose LPS infusion (100 ng LPS/24 h), the dark shaded area represents the period of increased infusion (250 ng LPS/24 h). The black dashed vertical lines show the timing of LPS bolus administration. The solid blue vertical lines show the timing of (5 mg/kg) Etanercept administration, which were 30 min before starting LPS infusion and 30 min before the first LPS bolus. Controls received an equivalent volume of vehicle (saline) during the infusion and bolus periods. Continuous physiological recordings were performed throughout the experimental period. Fetal preductal arterial blood was collected every morning starting from 30 min before the experiment until the day of post mortem. Additional blood samples were collected immediately before the LPS or saline boluses and 2 and 6 h after bolus infusions for measurement of cytokine levels

At 104 days, after 5–7 days postoperative recovery, fetuses were randomly allocated to receive an intravenous infusion of normal saline (*n* = 7), lipopolysaccharide (LPS; *n* = 8) dissolved in saline or Etanercept (2 doses of 5 mg/kg i.v.) dissolved in 4 mL normal saline, at a rate of 0.13 mL/min, 30 min before starting the LPS infusion and 30 min before the first LPS bolus (ET + LPS; *n* = 8). The study protocol is illustrated in Fig. 1. The rationale for choosing 5 mg/kg was guided by previous preclinical studies in rodents [25, 26]. The dose used in the rodent studies was converted to a human equivalent dose using FDA guidelines that account for the difference in body surface area between species [27]. This suggested that a human equivalent dose would be 2 mg/kg. In preliminary studies using our preclinical model of LPS-induced fetal inflammation, we compared 2 to 5 mg/kg. Assessment of neuroinflammation from these preliminary studies indicated a greater reduction in periventricular microgliosis after infusion of 5 mg/kg. Therefore, we chose 5 mg/kg for the present study.

LPS was infused at 100 ng/kg (50 ng/mL at 83.3 μl/h) for the first 24 h then 250 ng/kg/24 h (50 ng/mL at 207.5 μl/h) for the next 96 h. LPS boluses were infused as 1 μg LPS dissolved in 2 mL of saline (infusion rate 1 mL/min) at 48, 72, and 96 h after the start of infusion. This experimental model is most relevant to subclinical infection/inflammation with acute exacerbations, which is reported to be associated with adverse neurodevelopmental outcomes [28, 29]. Controls received the same volume of saline for infusions and boluses. Ten days after the start of infusions, sheep were killed by intravenous injection of an overdose (9 g) of pentobarbital sodium (Pentobarb 300, Chemstock international, Christchurch, New Zealand).

Fetal preductal arterial blood was collected every morning starting from 30 min before the experiment until the day of post mortem for pH, blood gases, (ABL 800, Radiometer, Copenhagen, Denmark), glucose, and lactate (model 2300, YSI, OH, USA).

### Fetal cytokine measurements

Additional blood samples were collected immediately before the LPS or saline boluses and 2 and 6 h after bolus infusions for measurement of cytokine levels using in-house enzyme-linked immunosorbent assays [13]. The time-points chosen for cytokine analysis were based on previous studies that used this experimental paradigm [12, 18]. Ovine-specific antibodies were used to measure fetal plasma for TNF and interleukin (IL)-6 (Epitope technologies, Melbourne, Australia), whereas bovine antibodies that cross-react with sheep were used to measure IL-10 (AbD Serotec, MorphoSys, Kidlington, UK). Standards were ovine recombinant TNF (range 0–10 ng/mL, detection sensitivity 0.354 ng/mL) and IL-6 (range 0–5 ng/mL, detection sensitivity 0.097 ng/mL; Protein Express, Cincinnati OH, USA), and recombinant bovine IL-10 (range 0–11 biological units (BU)/mL, detection sensitivity 0.086 BU/mL; supplied by Prof. G Entrican, Moredun Research Institute, Midlothian, Scotland). Internal quality controls were included in each assay and cytokine concentrations were within the detection limit in all samples. Etanercept reduces the bioactivity of circulating TNF but does not inhibit its cellular production [30], and we have previously shown that circulating TNF is upregulated in this experimental paradigm of fetal inflammation [18]. Therefore, to confirm circulating TNF levels were upregulated after LPS-exposure, we measured TNF in a sub-cohort (*n* = 3) of LPS and ET + LPS treated fetuses during the first 24 h after the first LPS bolus.

### Histopathology

**Fig. 2:**
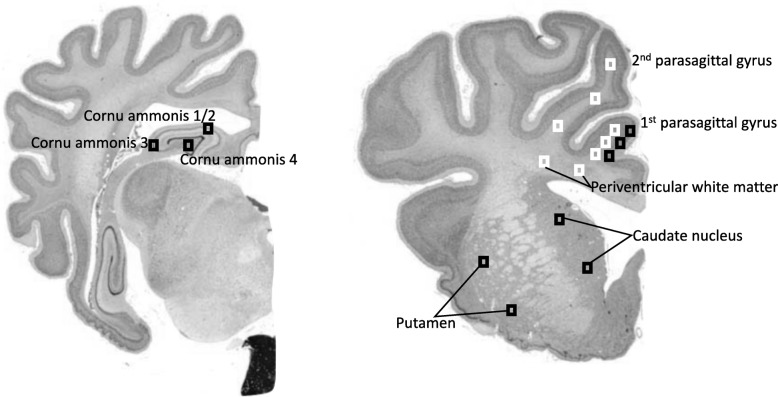
Schematic indicating fields sampled (regions of interest) for histological assessment. Left shows the cornu ammonis of the dorsal horn of the anterior hippocampus (CA 1–2, 3, 4) that was examined on sections taken 17 mm anterior to stereotaxic zero. Right shows brain regions of the forebrain used for analysis; these included the premotor cortex, caudate nucleus and putamen at the level of the mid-striatum, and periventricular and intragyral white matter from sections taken 23 mm anterior to stereotaxic zero. Black squares were sampled for assessment of neuronal survival within the first parasagittal gyri, caudate nucleus, and putamen. White squares were sampled for assessment of intragyral white matter within the first and second parasagittal gyri and the periventricular white matter. Image source: [31]

At post mortem (10 days after the start of infusions), fetal brains were perfusion fixed in situ with 10% phosphate-buffered formalin. Following removal from the skull, tissue was fixed for a further 5 days before processing and embedding using a standard paraffin tissue preparation. Brain slices were cut (10 μm thick) using a microtome (Leica Jung RM2035, Leica Microsystems, Albany, New Zealand). The cornu ammonis of the dorsal horn of the anterior hippocampus (CA 1–2, 3, 4) were examined on sections taken 17 mm anterior to stereotaxic zero (Fig. 2). Brain regions of the forebrain used for analysis included the premotor cortex, caudate nucleus, and putamen at the level of the mid-striatum, and periventricular and intragyral white matter from sections taken 23 mm anterior to stereotaxic zero. Slides were dewaxed in xylene, rehydrated in decreasing concentrations of ethanol, and then washed in 0.1 mol/L phosphate-buffered saline (PBS). Antigen retrieval was performed in citrate buffer (pH 6.0) using the pressure cooker technique in an antigen retrieval system (EMS Antigen 200 Retriever, Emgrid, Australia). Endogenous peroxidase quenching was performed by incubation in 0.1% H_2_O_2_ in methanol. Non-specific antigens were blocked using 3% normal goat serum. The sections were labeled with 1:200 mouse anti-NeuN (Chemicon International, Temecula, CA, USA), 1:200 rabbit anti-Olig-2 (Chemicon International; a marker of oligodendrocytes at all stages of the lineage) [32], 1:200 rabbit anti-Iba1 (Abcam, Hamilton, New Zealand), 1:200 mouse anti-GFAP (Abcam), 1:250 mouse anti-TNF (Bio-Rad, NSW, Australia), 1:200 rabbit anti-CNPase (Abcam), 1:200 rat anti-MBP (Merck, Darmstadt, Germany), and 1:200 mouse anti-Ki-67 (Dako, NSW, Australia) overnight at 4 °C. Sections were incubated in biotin-conjugated IgG (1:200, goat anti-rabbit or mouse; Vector Laboratories, Burlingame, CA, USA) for 3 h at room temperature. Sections were incubated in ExtrAvidin®-peroxidase (1:200, Sigma Aldrich, Auckland, New Zealand) for 2 h at room temperature and allowed to react with 3,3′-diaminobenzidine tetrahydrochloride (DAB; Sigma-Aldrich). The reaction was stopped by washing in phosphate-buffered saline prior to being dehydrated and mounted.

Neurons (NeuN), oligodendrocytes (Olig-2 and CNPase), myelination (MBP), proliferative (Ki-67) cells, microglia (Iba-1), and astrocytes (GFAP) were quantified at × 20 magnification and TNF-positive cells were quantified at × 40 magnification, using a Nikon 80i light microscope and NIS elements BR 4.0 software (Nikon Instruments Inc., Melville, NY, USA). Microglia (Iba-1-positive cells) showing both ramified and amoeboid morphology were included in our assessment. Astrocytes were quantified by counting the density of GFAP positive cell bodies. Astrocytes that were deemed morphologically normal (GFAP-positive cells displaying a ramified morphology) and reactive (GFAP-positive cells with fewer and thicker retracted processes), as described by Liddelow et al. [33] were included. The area fraction of NeuN and MBP immunoreactivity was determined with a standard intensity threshold using ImageJ software (NIH, Bethesda, MD, USA). Average scores from both hemispheres from two adjacent sections were calculated for each region and the region of interest was randomized within each area. All imaging and cell counts were performed by an assessor (RG) who was blinded to the treatment.

### Data analysis and statistics

Off-line physiological data analysis was performed using Labview based customized programs (LabVIEW for Windows, National Instruments Inc.). Carotid and femoral vascular conductance were calculated as mean blood flow/MAP. To facilitate the assessment of cardiovascular impairment after LPS-exposure, we classified hypotension as a fall in MAP of ≥ 5 mmHg (equivalent to a fall of ≥ 2 standard deviations from baseline MAP) after the first LPS bolus [34]. The proportion of immature/mature oligodendrocytes within the intragyral and periventricular white matter was calculated as the ratio of CNPase to Olig-2-positive cells.

Statistical analysis was undertaken using SPSS (v22 SPSS, Chicago, IL, USA) and Sigmaplot software (v12 Systat Software, San Jose, CA, USA). Between and within-group comparisons of fetal pH, blood gases, glucose, lactate, and physiological data were performed by two-way repeated measures ANOVA. Physiological data for the baseline, infusion, bolus, and recovery periods were analyzed as separate time periods. When statistical significance was found between groups or between group and time, post hoc comparisons were made using a Fisher’s least significant difference test. Between groups comparison of neuropathological data were performed using a two-way ANOVA, followed by Fisher’s least significant difference post hoc test when significance was found between groups or region and group [35]. If there was an effect of region and group, the effect of the group was assessed for each region separately. Mann-Whitney *U* tests were used for testing non-parametric data. Statistical significance was accepted when *P* < 0.05.

## Results

### Baseline period

Before LPS-exposure, baseline MAP, FHR, carotid and femoral blood flows, EEG amplitude and spectral edge frequency, nuchal EMG, blood gases, and glucose and lactate concentrations were within the normal range by our laboratory standards and did not differ between groups.

### Fetal biochemistry

There were no significant differences in fetal arterial biochemistry parameters between groups during the baseline and low dose infusion periods (Table 1). pH was lower in the LPS group at 2 h after the first LPS bolus compared to controls (*P* < 0.05). PCO_2_ was higher in the LPS group at 6 h after the first LPS bolus compared to controls (*P* < 0.05). Lactate was higher in the LPS group at 2 and 6 h after the first LPS bolus compared to both control and ET + LPS groups (*P* < 0.01, respectively).

**Table 1:** Arterial pH, blood gases, glucose, and lactate values

		Baseline	Before bolus 1	Bolus 1 + 2 h	Bolus 1 + 6 h	Before bolus 2	Bolus 2 + 2 h	Bolus 2 + 6 h	Before bolus 3	Bolus 3 + 2 h	Bolus 3 + 6 h	Recovery
pH	Saline	7.33 ± 0.01	7.33 ± 0.01	7.34 ± 0.01	7.33 ± 0.01	7.33 ± 0.01	7.33 ± 0.01	7.33 ± 0.01	7.32 ± 0.01	7.33 ± 0.01	7.32 ± 0.01	7.33 ± 0.01
	LPS	7.34 ± 0.01	7.32 ± 0.01	7.30 ± 0.01*	7.32 ± 0.01	7.33 ± 0.01	7.31 ± 0.01	7.34 ± 0.00	7.33 ± 0.01	7.33 ± 0.00	7.33 ± 0.01	7.34 ± 0.01
	ET + LPS	7.34 ± 0.01	7.32 ± 0.01	7.32 ± 0.01	7.32 ± 0.01	7.31 ± 0.01	7.31 ± 0.01	7.32 ± 0.01	7.32 ± 0.01	7.33 ± 0.01	7.33 ± 0.01	7.32 ± 0.02
PCO2	Saline	47.1 ± 1.0	48.8 ± 1.6	47.2 ± 1.7	44.4 ± 3.9	48.4 ± 1.3	49.0 ± 1.9	48.1 ± 1.2	48.1 ± 1.5	46.1 ± 1.8	48.9 ± 1.7	50.6 ± 1.9
	LPS	47.1 ± 1.6	46.8 ± 1.9	48.8 ± 2.4	51.9 ± 1.2*	48.1 ± 2.3	48.1 ± 1.4	50.7 ± 1.4	46.1 ± 1.8	45.7 ± 2.0	47.6 ± 1.1	46.5 ± 0.7
	ET + LPS	44.1 ± 1.9	45.0 ± 2.4	44.4 ± 1.8	51.3 ± 1.9	46.0 ± 2.0	47.6 ± 2.3	49.6 ± 2.4	44.9 ± 1.9	44.9 ± 1.6	46.1 ± 2.1	46.7 ± 1.9
PO2	Saline	22.6 ± 1.12	23.7 ± 1.2	23.8 ± 1.1	22.1 ± 1.4	23.9 ± 1.4	23.6 ± 1.5	22.1 ± 1.3	24.2 ± 1.4	22.9 ± 1.1	21.8 ± 1.2	24.0 ± 1.1
	LPS	25.3 ± 1.5	27.0 ± 1.8	26.2 ± 1.9	22.2 ± 2.0	26.0 ± 1.8	25.0 ± 1.6	23.0 ± 1.8	26.7 ± 1.7	26.0 ± 1.5	25.3 ± 1.7	28.2 ± 2.3
	ET + LPS	27.0 ± 1.1	27.8 ± 0.8	27.5 ± 1.1	23.0 ± 1.1	27.2 ± 0.7	25.5 ± 0.6	20.9 ± 2.0	27.6 ± 0.8	26.3 ± 0.8	28.1 ± 1.3	27.6 ± 0.8
Glucose	Saline	0.7 ± 0.1	0.7 ± 0.1	0.8 ± 0.1	0.8 ± 0.1	0.7 ± 0.0	0.8 ± 0.0	0.7 ± 0.1	0.7 ± 0.0	0.7 ± 0.1	0.8 ± 0.1	0.7 ± 0.0
	LPS	0.9 ± 0.1	0.9 ± 0.1	0.9 ± 0.1	0.8 ± 0.0	0.9 ± 0.1	0.8 ± 0.1	0.8 ± 0.0	0.9 ± 0.1	0.8 ± 0.1	0.9 ± 0.1	0.9 ± 0.1
	ET + LPS	0.8 ± 0.1	0.9 ± 0.1	0.9 ± 0.1	0.9 ± 0.1	0.9 ± 0.1	0.9 ± 0.1	0.9 ± 0.1	0.9 ± 0.1	1.0 ± 0.1	1.0 ± 0.1	1.0 ± 0.1
Lactate	Saline	0.7 ± 0.1	0.8 ± 0.0	0.8 ± 0.0	0.9 ± 0.0	0.8 ± 0.1	0.8 ± 0.1	0.9 ± 0.1	0.8 ± 0.0	0.8 ± 0.1	0.9 ± 0.1	0.8 ± 0.1
	LPS	0.9 ± 0.1	0.8 ± 0.1	1.5 ± 0.2*#	2.5 ± 0.6*#	0.8 ± 0.1	1.2 ± 0.2	1.3 ± 0.3	0.7 ± 0.1	0.8 ± 0.1	0.8 ± 0.1	0.7 ± 0.1
	ET + LPS	0.7 ± 0.1	0.7 ± 0.1	0.9 ± 0.1	1.1 ± 0.1	0.7 ± 0.1	0.9 ± 0.0	1.1 ± 0.2	0.7 ± 0.1	0.8 ± 0.1	0.8 ± 0.1	0.8 ± 0.1

Data are means ± SEM. *LPS* lipopolysaccharide, *ET* Etanercept, *PCO2* partial pressure of arterial carbon dioxide, *PO2* partial pressure of arterial oxygen. *P < 0.05 vs control, #*P* < 0.05 vs ET + LPS

### Plasma cytokines

**Fig. 3:**
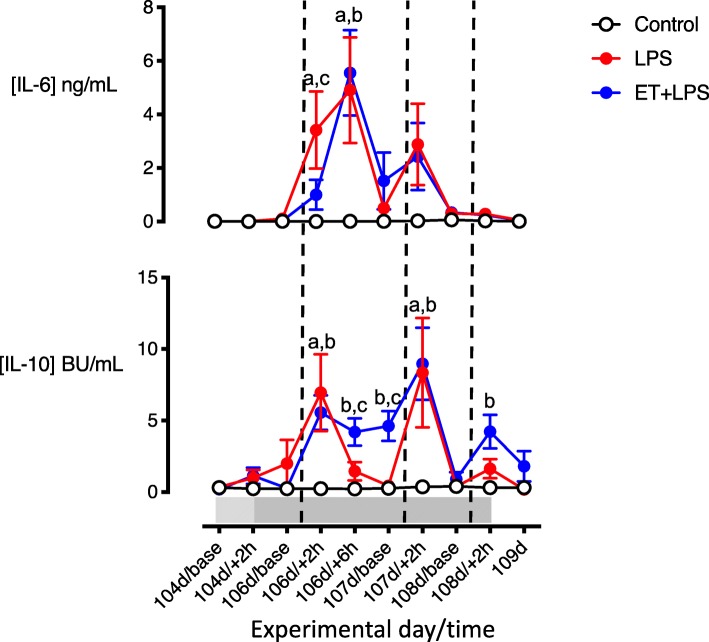
Time-course of changes in circulating interleukins. Time-course of changes in circulating interleukin (IL)-6 and IL-10 in the control (open circles, *n* = 7), LPS (red circles, *n* = 8) and ET + LPS (blue circles, *n* = 8) groups. The light shaded area represents the period of low dose LPS infusion, the dark shaded area represents the period of increased infusion. The dashed vertical lines show the timing of LPS/saline bolus administration. Data are hourly means ± SEM. LPS, lipopolysaccharide, ET, Etanercept. a = *P* < 0.05 LPS vs. control, b = *P* < 0.05 ET + LPS vs. control, c = *P* < 0.05 LPS vs. ET + LPS

Circulating TNF levels did not differ between LPS-exposed groups before the first LPS bolus. In both LPS groups, TNF levels peaked at + 2 h after the first LPS bolus (LPS = 9.2 ± 2.2 vs. ET + LPS = 9.5 ± 0.9 ng/mL, *P* < 0.05 vs. baseline) and remained elevated at + 6 h after the first LPS bolus (LPS = 5.0 ± 1.6 vs. ET + LPS = 5.2 ± 0.3 ng/mL, *P* < 0.05 vs. baseline). Plasma TNF levels returned to baseline levels within 24 h after the first LPS bolus. Before the first LPS bolus, plasma IL-6 and IL-10 levels did not differ between groups. At 2 and 6 h after the first LPS bolus (106 days + 2 h and + 6 h), circulating IL-6 was increased in the LPS group compared to controls (*P* < 0.05). In ET + LPS-treated fetuses, plasma IL-6 was lower compared to the LPS group at 2 h after the first LPS bolus (106 days + 2 h, *P* < 0.05). Two hours after the first and second LPS boluses (106 days + 2 h and 107 d + 2 h, respectively), plasma IL-10 was increased in LPS and ET + LPS groups compared to control (*P* < 0.05). In ET + LPS-treated fetuses, plasma IL-10 levels were higher compared to LPS and control groups at 6 h and 24 h after the first LPS bolus (106 days + 6 h and 107 days baseline, Fig. 3). At + 2 h after the third LPS bolus (108 days + 2 h), plasma IL-10 was higher in ET + LPS-treated fetuses compared to control.

### Mean arterial pressure

There was no significant effect of low-dose LPS infusion on MAP. In LPS and ET + LPS-exposed groups, 2/8 fetuses did not develop hypotension (defined by a fall in MAP ≥ 5 mmHg from baseline) after the first LPS bolus. After the first LPS bolus (48 h), MAP was increased in the LPS group compared to ET + LPS treated fetuses at 50 h (2 h after the first LPS bolus, *P* < 0.05). MAP was reduced in LPS and ET + LPS-treated fetuses compared to control between 52 and 62 h (4–15 h after the first LPS bolus, *P* < 0.05). MAP progressively recovered to baseline in the LPS and ET + LPS groups between 58 and 64 h (10 and 16 h after the first LPS bolus). After the second LPS bolus, MAP was lower in the LPS group compared to controls between 76 and 82 h (4–12 h after the second LPS bolus, *P* < 0.05), and recovered to baseline by approximately 85 h (6 h after the MAP nadir). In ET + LPS-treated fetuses, the second LPS bolus had no significant effect on MAP. In the LPS and ET + LPS-treated groups, there were no differences in MAP compared to controls after the third LPS bolus. There was no difference in MAP between groups during the recovery period.

### Fetal heart rate

There was no significant effect of the low dose LPS infusion on FHR. After the first LPS bolus, FHR was increased in the LPS group compared to controls between 50 and 61 h (2–13 h after the first LPS bolus, *P* < 0.05). In ET + LPS-treated fetuses, FHR did not differ from control and was lower compared to the LPS group at 52 and 57–59 h (4 and 9–11 h after the first LPS bolus, respectively; *P* < 0.05). In the LPS group, FHR progressively recovered by 66 h (18 h after the first LPS bolus). After the second and third LPS boluses, FHR was increased in the LPS group compared to controls from 75 to 81 h (3–9 h after the second LPS bolus, *P* < 0.05) and from 100 to 104 h (4–8 h after the third LPS bolus, *P* < 0.05). In ET + LPS-treated fetuses, FHR did not differ from control after the second and third LPS boluses and was lower from 77 to 79 h (5–7 h after the second LPS bolus, *P* < 0.05 compared to the LPS group), and from 4 to 8 h after the third LPS bolus (100–104 h, *P* < 0.05). During recovery, FHR did not differ between groups.

### Carotid and femoral arterial blood flows

**Fig. 4:**
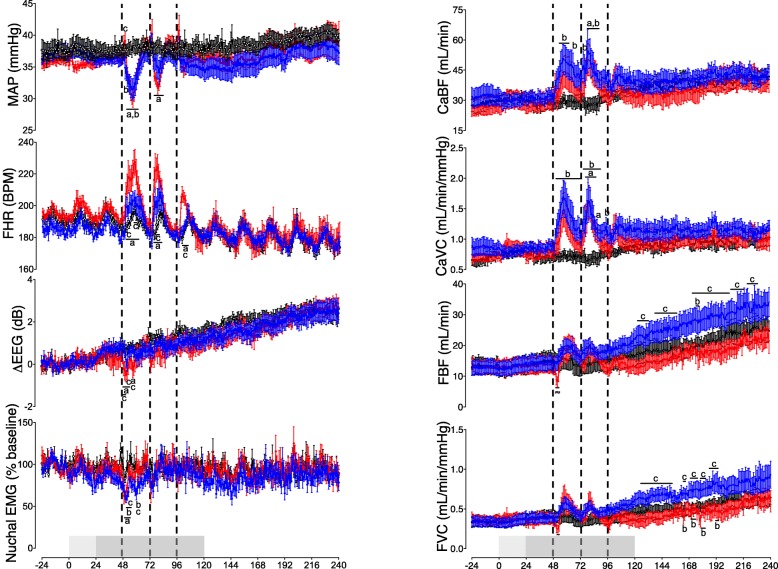
Cardiovascular and cerebrovascular changes. Mean arterial pressure (MAP), carotid artery blood flow (CaBF), fetal heart rate (FHR), carotid artery vascular conductance (CaVC), EEG power, femoral artery blood flow (FBF), nuchal electromyography (EMG), and femoral artery vascular conductance (FVC) in the control (open circles, *n* = 7), LPS (red circles, *n* = 8) and ET + LPS (blue circles, *n* = 8) groups. The light shaded area represents the period of low dose LPS infusion, and the dark shaded area represents the period of increased infusion. The dashed vertical lines show the timing of LPS/saline bolus administration. Data are hourly means ± SEM. LPS, lipopolysaccharide, ET, Etanercept; a = *P* < 0.05 LPS vs. control, b = *P* < 0.05 ET + LPS vs. control, c = *P* < 0.05 LPS vs. ET + LPS

During the baseline and low-dose infusion periods, carotid and femoral arterial blood flows and vascular conductance did not differ between groups (Fig. 4). After the first LPS bolus, carotid arterial blood flow was higher in ET + LPS-exposed fetuses between 53 and 61 h and at 66 h (5–13 h and 18 h after the first LPS bolus, respectively; *P* < 0.05 compared to controls). After the second LPS bolus, carotid arterial blood flow was higher than controls in the ET + LPS group at 75 h (3 h after the second LPS bolus, *P* < 0.05). Carotid arterial blood flow was increased in LPS and ET + LPS-treated groups from 78 to 87 h (6–15 h after the second LPS bolus, *P* < 0.05 vs. control).

After the first LPS bolus, carotid arterial vascular conductance was higher in ET + LPS exposed fetuses between 51 and 71 h (3–23 h after the first LPS bolus, *P* < 0.05 vs. control, Fig. 4). After the second LPS bolus, carotid arterial vascular conductance was higher in the ET + LPS group compared to controls from 74 to 89 h and at 95 h (2–17 h and 23 h after the second LPS bolus, *P* < 0.05). In the LPS group, carotid arterial vascular conductance was higher than controls from 77 to 85 h and at 87 h (5–13 h and 15 h after the second LPS bolus, *P* < 0.05). For the remainder of the experimental period, there were no differences in carotid arterial blood flow or vascular conductance between groups.

Between 51 and 52 h (3–4 h after the first LPS bolus), femoral blood flow and vascular conductance were not significantly lower in the LPS group compared to the control and ET + LPS groups (*P* < 0.07). During the recovery period, FBF and FVC were higher in the ET + LPS-treated fetuses compared to the LPS group between 120 and 225 h (*P* < 0.05). Thereafter, there were no differences in femoral blood flow or vascular conductance between groups.

### EEG activity

During the study, there were no significant differences in EEG spectral edge frequency between the groups. During the baseline and low-dose infusion periods, EEG power did not differ between groups. EEG power was lower in the LPS group compared to the control and ET + LPS groups (*P* < 0.05) between 49 and 53 h and at 57 h (1–5 and 9 h after the first LPS bolus). Thereafter, there were no significant differences in EEG power between groups.

### Nuchal EMG activity

During the baseline and low-dose infusion periods, nuchal EMG activity did not differ between groups. After the first LPS bolus, nuchal EMG activity was reduced in the LPS and ET + LPS groups between 50 and 54 h (2–6 h after the first LPS bolus, *P* < 0.05 vs. control). In ET + LPS-treated fetuses, nuchal EMG activity was lower compared to the LPS group at 53 and 60 h (5 and 12 h after the first LPS bolus, *P* < 0.05) and was lower than controls at 60 h (*P* < 0.05).

### Post mortem findings

There were no significant differences in body weight, brain weight, or the ratio of males to females between the groups (Table 2).

**Table 2:** Fetal body weight, brain weight, and sex

	Body weight (kg)	Brain weight (g)	Sex (M:F)
Saline	2.02 ± 0.07	31.7 ± 1.1	4:4
LPS	2.04 ± 0.11	30.5 ± 1.2	5:3
ET + LPS	2.00 ± 0.13	31.8 ± 1.8	2:6

Data are means ± SEM. *LPS* lipopolysaccharide, *ET* Etanercept

### Histopathology

**Fig. 5:**
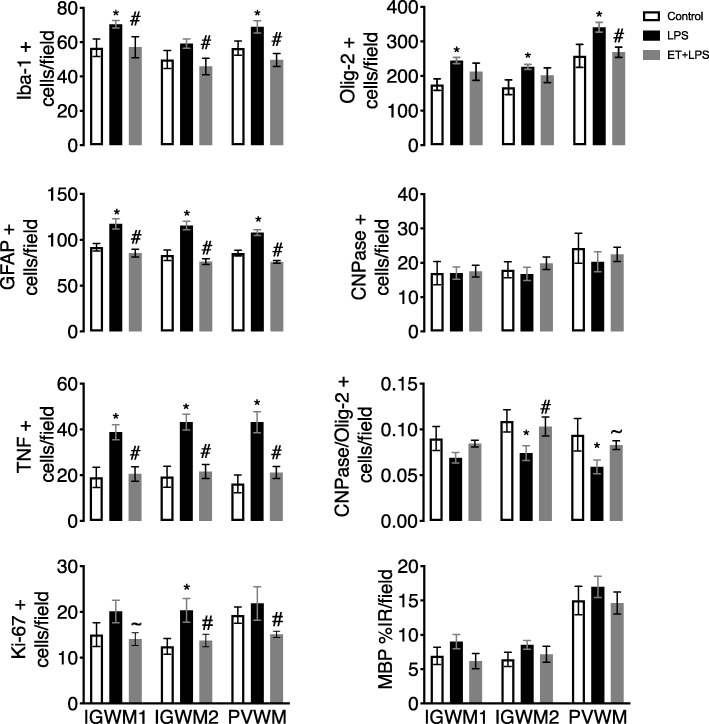
White matter immunohistochemistry. Iba-1, Olig-2, GFAP, TNF, CNPase, Ki-67 positive cell counts, the proportion of mature CNPase oligodendrocytes (CNPase/Olig-2-positive cells), and the percentage area of MBP-positive staining in intragyral and periventricular white matter (IGWM and PVWM, respectively) in the control (open bars, *n* = 7), LPS (black bars, *n* = 8) and ET + LPS (gray bars, *n* = 8) groups. Data are means ± SEM. LPS, lipopolysaccharide, ET, Etanercept. *P < 0.05 vs. control, #*P* < 0.05 vs. LPS, ~*P* < 0.07 vs. LPS

**Fig. 6:**
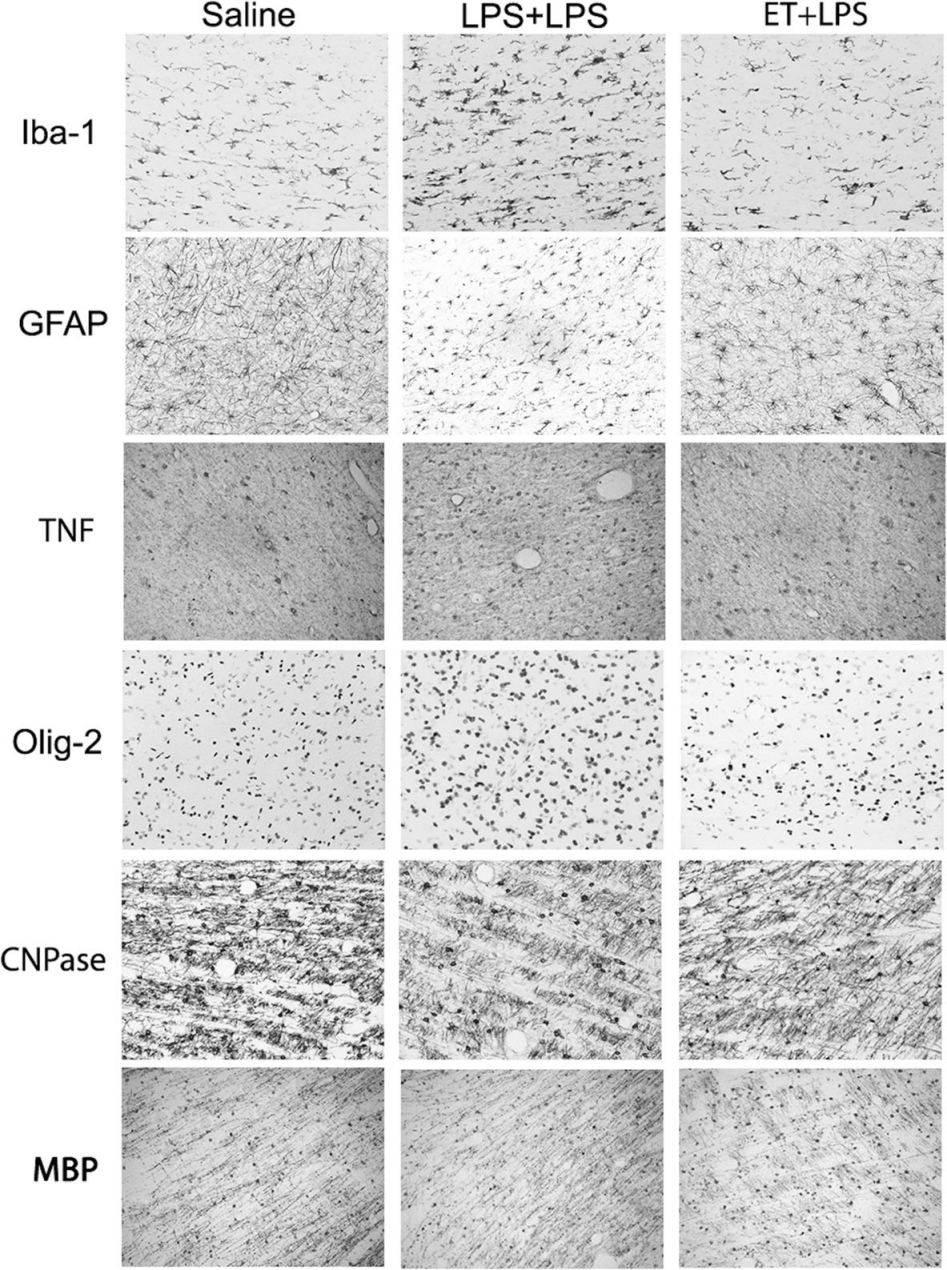
Photomicrographs of white matter tracts. Representative photomicrographs showing Iba-1, GFAP, TNF, Olig-2, CNPase, and MBP-positive staining in the periventricular white matter tracts. LPS, lipopolysaccharide, ET, Etanercept. Scale bar is 200 μm

The number of Iba-1, GFAP, and TNF-positive cells was increased in the intragyral and periventricular white matter tracts in the LPS group compared to controls (*P* < 0.05, Figs. 5 and 6). In ET + LPS-treated fetuses, the number of Iba-1, GFAP, and TNF-positive cells was reduced in both white matter tracts compared to the LPS group (*P* < 0.05).

The number of Ki-67-positive cells was increased in the second intragyral white matter tract in the LPS group (*P* < 0.05 compared to controls). In ET + LPS-treated fetuses, the number of Ki-67-positive cells was reduced compared to the LPS group in the second intragyral white matter tract and periventricular white matter (*P* < 0.05). Post hoc analysis revealed a statistically borderline reduction in Ki-67-positive cells in the first intragyral white matter tract of the ET + LPS-treated group (*P* < 0.07 vs. LPS).

In the intragyral and periventricular white matter, the number of Olig-2-positive cells was increased in the LPS group (*P* < 0.05, compared to controls, Figs. 5 and 6). In the ET + LPS group, the number of Olig-2-positive cells was reduced compared to the LPS group within the periventricular white matter (*P* < 0.05), and did not differ from controls. The number of CNPase-positive cells did not differ between groups in the intragyral and periventricular white matter tracts. In LPS exposed fetuses, the proportion of immature and mature oligodendrocytes (i.e., CNPase + cells/Olig-2 + cells) was reduced in the second intragyral white matter tract and the periventricular white matter compared to controls (*P* < 0.05), and were normalized in the ET + LPS group in these regions. The % area fraction of MBP staining in the intragyral and periventricular white matter tracts did not differ between groups.

**Fig. 7:**
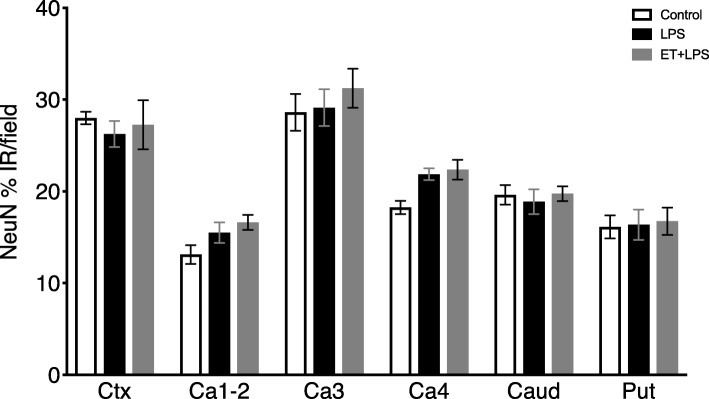
Neuronal survival in cortical and subcortical brain regions. Anti-neuronal nuclei (NeuN) monoclonal antibody percent area of immunoreactivity (%IR) in the premotor cortex (Ctx), cornu ammonis (CA) 1–2, 3 and 4, caudate nucleus (Caud), and putamen (Put) in control (open bars, *n* = 7), LPS (black bars, *n* = 8) and ET + LPS (gray bars, *n* = 8) groups. Data are means ± SEM. LPS, lipopolysaccharide, ET, Etanercept

There were no significant differences in the area fraction of NeuN-positive staining between groups in the cortex, hippocampus, and striatum (Fig. 7).

## Discussion

This study shows that TNF inhibition during prolonged systemic, LPS-induced inflammation in preterm fetal sheep prevented gliosis and normalized proliferation and total oligodendrocyte counts in the large white matter tracts, but did not affect numbers of immature/mature oligodendrocytes or myelin expression. The reduction in neuroinflammation was associated with reduced pro-inflammatory and increased anti-inflammatory cytokine production, and improved recovery of EEG power and cardiovascular function after LPS.

Clinically, systemic upregulation of TNF is independently associated with increased risk of short- and long-term neurodevelopmental impairment after preterm birth [14, 15, 36]. Furthermore, increased TNF expression has been detected throughout white and gray matter regions in preterm infants with periventricular leukomalacia [10, 11]. These data demonstrate a strong association between elevated systemic and local TNF production, and perinatal brain injury. However, it remains unknown whether this association is causal. Critically, this study shows that TNF is likely to be one contributor to white matter inflammation. The present data in a relevant large animal model are highly consistent with recent evidence for benefit with TNF inhibition after acute LPS exposure in the neonatal rat [22] and suggest that targeted systemic inhibition may have therapeutic value.

Consistent with previous work from our laboratory and others, high-dose LPS boluses were associated with a systemic inflammatory response, as shown by elevated cytokine levels (TNF, IL-6, and IL-10). These inflammatory responses were associated with peripheral vasodilation, hypotension, and tachycardia in LPS-exposed fetuses, similar to previous studies [16–18]. Exposure to a low-dose infusion of LPS induced tolerance to subsequent high-dose LPS exposures. This well-known phenomenon is partly mediated by the reprogramming of the innate immune system. For example, in human and fetal sheep monocytes, repeated LPS exposure is associated with decreased production of pro-inflammatory cytokines and downregulation of the LPS receptor CD14 [37–39]. Consistent with these findings, repeated LPS exposure in preterm fetal and neonatal sheep is associated with attenuation of the systemic inflammatory response [12, 13, 17, 34, 40].

In the present study, TNF inhibition with Etanercept significantly but transiently reduced circulating IL-6 levels, which are mediated by TNF [41], at 2 h after the first LPS bolus, and was associated with prolonged upregulation of the anti-inflammatory cytokine IL-10. However, this regime did not attenuate subsequent changes in IL-6, and in particular not the peak at 6 h after the LPS bolus. To the best of our knowledge, the temporal profile of circulating cytokines has not been previously assessed in the setting of Etanercept and preterm systemic inflammation. Our data now suggest that higher doses of Etanercept may be needed to further improve outcomes during prolonged exposure to infection/inflammation.

Nevertheless, the transient reduction in circulating IL-6 in the ET + LPS group likely reflects reduced TNF bioactivity after Etanercept treatment, as previously shown [42]. Furthermore, the prolonged elevation of circulating IL-10 is supported by in vitro and in vivo data showing that TNF inhibition can augment IL-10 production by modifying T cell function [25, 43]. Critically, IL-10 has been shown to be neuroprotective in preclinical models of perinatal inflammation [44]. Circulating TNF levels were not reduced after Etanercept treatment, consistent with its known mechanism of action, through reduced bioactivity of TNF [30, 42]. Further, because of the similar binding site, TNF immunoassays typically cross-react with Etanercept and so circulating TNF levels are not a reliable indicator of biologically active TNF [45]. By contrast, Etanercept was shown to reduce circulating TNF levels in a rodent model of adult traumatic brain injury, which produced a relatively mild level of systemic inflammation (peak plasma TNF 20 pg/mL measured 72 h after injury) [25] relative to our experimental paradigm of fetal systemic inflammation (peak plasma TNF 9000 pg/mL). This suggests the magnitude of systemic inflammation may influence the capacity of Etanercept to affect absolute TNF levels.

In preterm neonates, elevated cord blood levels of TNF are strongly associated with moderate transient suppression of EEG power and poor neurodevelopmental outcomes, such as cerebral palsy [14]. Similarly, in the present study, we observed a transient moderate reduction in EEG and nuchal EMG activity (reflecting reduced neural activity and fetal movement, respectively) after the first high-dose LPS bolus. The suppression of EEG power and fetal movement, which may reflect inhibition of synaptic activity due to increased local cytokine production [46–48], were associated with significantly elevated circulating IL-6 levels and neuroinflammation that are characteristic of preterm brain injury. Further, both inflammation and hypotension can trigger active EEG suppression through the release of inhibitory neuromodulators, such as adenosine and neurosteroids such as allopregnanolone [49–51]. Although in the present study, systemic hypotension and EEG suppression were not associated with reduced carotid artery perfusion, there was an increase in circulating lactate within the first 6 h after the first high dose LPS bolus, suggesting impaired oxidative phosphorylation at that time.

These data raise the possibility that fetal inflammation leads to a shift towards higher cerebral metabolic demand, and in turn, increases the susceptibility to hypoxic-ischemic brain injury in preterm infants. This concept is supported by studies in preterm infants that showed increased cerebral oxygen extraction after intrauterine inflammation, and a greater incidence of peri/intraventricular hemorrhage in the first 24 h of life [52]. However, further studies of cerebral metabolism and oxygenation during intrauterine inflammation are required to determine if the effects of systemic inflammation on EEG activity reflect actively mediated suppression or passive anoxic depolarization. Etanercept treatment was associated with a reduction in both the magnitude and duration of EEG suppression, as well as increased carotid artery perfusion, compared to controls, suggesting improved cerebral oxygen supply and metabolism. Furthermore, a small but significant delayed suppression of nuchal EMG activity was observed in Etanercept-treated fetuses compared to control. This may reflect a delayed effect of LPS-induced inflammation on fetal movement following treatment. Consistent with improved fetal tissue oxygenation, circulating lactate levels in the ET + LPS-treated fetuses did not differ from controls. Thus, these data suggest that increased circulating TNF plays an important role in mediating EEG suppression during fetal inflammation.

Consistent with previous studies, fetal LPS-induced inflammation was associated with increased numbers of microglia, astrocytes, and TNF-positive staining within the intragyral and periventricular white matter tracts [12, 13]. This was accompanied by increased total (Olig-2 + ve) oligodendrocytes and cell proliferation within the intragyral and periventricular white matter. There was no effect on total myelination, as shown by no differences in total numbers of immature and mature (CNPase-positive) oligodendrocytes or MBP immunoreactivity between the groups. This combination suggests an increase in oligodendrocyte precursor proliferation in LPS-exposed fetuses. Similarly, we found no overt neuronal loss after LPS exposure, as shown by no differences in cortical and subcortical NeuN immunoreactivity between the groups. Pathologically, this is highly consistent with the common clinical finding in preterm infants of diffuse white matter injury without overt neuronal loss [53, 54].

The mechanism of the apparent increase in oligodendrocyte precursors after LPS, and subsequent correction after Etanercept infusion, is unclear. The increase in astrocytes and microglia after LPS is consistent with a response to injury. Consistent with this hypothesis, in the P3 rat, acute LPS exposure was associated with apoptosis of oligodendrocyte precursors [22]. Alternatively, there is some evidence that inflammation itself can promote oligodendrocyte proliferation [55]. Further studies are now needed to understand whether this response affects long-term brain development.

Etanercept treatment during LPS-induced fetal inflammation reduced numbers of microglia, astrocytes, TNF-positive cells, and cell proliferation within the intragyral and periventricular white matter compared to LPS plus vehicle. This reduction in gliosis and cell proliferation in the ET + LPS-treated group was associated with normalization of total numbers of oligodendrocytes, suggesting reduced proliferation of pre-oligodendrocytes, but no effect on numbers of immature and mature oligodendrocytes (CNPase +ve cells) or myelin expression. These data are consistent with evidence that systemic cytokines such as TNF and IL-6 can cross the blood-brain barrier and activate cerebral microglia and astrocytes [56, 57].

Etanercept is a Food and Drug Administration-approved fusion protein that consists of the extracellular ligand-binding portion of the human p75 TNF receptor linked to the Fc portion of the human IgG1 molecule. It binds specifically to TNF to block its interaction with cell surface TNF receptors and reduce the pro-inflammatory function of the TNF protein. It has a molecular weight of 150 kDa, and so it is unlikely to penetrate the blood-brain barrier [42]. Nevertheless, murine studies have shown limited penetration of LPS, which has a molecular weight of 50–100 kDa, across the blood-brain barrier after systemic injection [58]. Therefore, we speculate that a transient reduction in circulating IL-6 levels and augmented production of the anti-inflammatory cytokine IL-10 could be potential mechanisms by which Etanercept attenuated white matter inflammation, as shown by reduced numbers of microglia and astrocytes in Etanercept+LPS-treated fetuses compared to the vehicle+LPS-treated group.

Etanercept infusion reduced the severity of hypotension after the second LPS bolus. TNF is capable of upregulating inducible nitric oxide synthase (iNOS), which is a potent vasodilator [59, 60]. The upregulation of iNOS plays a key role in vasodilation during septicemia and has been demonstrated after LPS-exposure in fetal sheep [16]. By contrast, reduced NOS activity inhibits LPS-induced vasodilation [61, 62]. Thus, the present data raises the possibility that TNF inhibition may have limited NO-mediated vasodilation or improved endothelial sensitivity to circulating vasoactive agents. However, further studies are needed to confirm this potential mechanism in the setting of preterm systemic inflammation.

Etanercept treatment also reduced the magnitude of tachycardia after high dose LPS-exposure. We have previously shown that fetal heart rate variability is not increased during the peak of tachycardia after LPS injection [18]. Thus, it is likely that the increase in FHR was mediated by LPS-induced catecholamine release, consistent with the effects of LPS and septic shock [63, 64]. In adults, TNF inhibition is associated with reduced catecholamine production [65]. This raises the possibility that increased beta-adrenergic activity led to tachycardia during high-dose LPS exposure. Collectively, the moderately higher carotid artery blood flow combined with a lower FHR in the Etanercept-treated fetuses compared to the vehicle + LPS group suggests an increase in stroke volume, possibly due to improved cardiac contractility. Supporting these data, systemic TNF inhibition has been associated with improved cardiac contractility in adult pigs exposed to systemic LPS [66]. During the recovery period, femoral blood flow and vascular conductance were higher in Etanercept-treated fetuses compared to the LPS + vehicle group. Furthermore, we observed select time-points when femoral blood flow and vascular conductance were higher in Etanercept-treated fetuses compared to control. Consistent with these data, Etanercept has been shown to reduce peripheral vascular resistance and increase peripheral blood flow by increasing endothelial vasoreactivity in patients with advanced heart failure [67].

Understandably, the dosing and route of administration used in this study are different from clinical protocols for Etanercept to treat chronic inflammation. This largely reflects that the preterm fetal sheep (1 kg) is much smaller than children or adult humans. Current protocols recommend subcutaneous doses of up to 50 mg/week in pediatric and adult populations. The dosing protocol chosen for this study consisted of two 5 mg doses, given 48 h apart (i.e., before inducing inflammation via chronic LPS infusion and the first LPS bolus, see Fig. 1), resulting in a total dose of 10 mg/week; this is relatively larger than typical clinical protocols, but highly consistent with previous studies in small animals [22]. Subcutaneous administration of Etanercept is slow release [68]. Given the rapid induction of inflammation with intravenous LPS infusion, we chose to administer Etanercept intravenously instead of subcutaneously.

One of the key translational considerations for potential neuroprotectants is when to treat [69]. In the present study, we gave the first dose of Etanercept immediately before LPS exposure. The rationale was to establish proof of principle that systemic TNF inhibition can alleviate neuroinflammation and injury. Given that circulating TNF levels peak within the first 2 h of high-dose LPS [18], it is likely that early detection and treatment will be important. However, it is important to appreciate that that in current practice it is unlikely that inflammation (infectious or sterile) can be detected and treated as soon as it begins, and it remains a formidable challenge to initiate experimental interventions within the first 6 h after birth [69]. Thus, the present study supports further investigation to determine the window of opportunity for treating inflammation-induced brain injury.

## Conclusions

In conclusion, administration of the soluble TNF antagonist, Etanercept, to preterm fetal sheep during inflammation prevented microgliosis, astrogliosis, and normalized neural TNF expression and proliferation, but did not affect myelination of the white matter tracts, 10 days after the start of LPS infusions. Notably, although Etanercept attenuated the early increase in IL-6 2 h after the first bolus of LPS, it had no effect on subsequent IL-6 levels. These data suggest a possible neuroprotective role for TNF inhibition in preterm infants exposed to inflammation during the perinatal period but higher doses of Etanercept may be needed during prolonged infection/inflammation. Based on these data, further translation preclinical studies are needed to evaluate the optimal dosing regimen and the efficacy of delayed administration to assess the potential for TNF inhibition to improve outcomes after preterm birth.

## Data Availability

The datasets used during the current study are available from the corresponding author on reasonable request.

## Abbreviations

CaBF: Carotid arterial blood flow
CaVC: Carotid arterial vascular conductance
CNPase: 2′,-3′-Cyclic-nucleotide 3′-phosphodiesterase
EEG: Electroencephalogram
FBF: Femoral arterial blood flow
FHR: Fetal heart rate
FVC: Femoral arterial vascular conductance
GFAP: Glial fibrillary acidic protein
Iba-1: Ionized calcium-binding adapter molecule-1
IGWM: Intragyral white matter
IL: Interleukin
LPS: Lipopolysaccharide
MAP: Mean arterial blood pressure
MBP: Myelin basic protein
Olig2: Oligodendrocyte transcription factor-2
PBS: Phosphate-buffered saline
PVWM: Periventricular white matter
TNF: Tumor necrosis factor
